# Novel Model to Predict the Prognosis of Patients with Stage II–III Colon Cancer

**DOI:** 10.1155/2020/8812974

**Published:** 2020-11-22

**Authors:** Yansong Xu, Fangfang Liang, Yi Chen, Zhen Wang, Huage Zhong, Weizhong Tang

**Affiliations:** ^1^Guangxi Clinical Research Center for Colorectal Cancer, Nanning, Guangxi Zhuang Autonomous Region 530021, China; ^2^Department of Gastrointestinal Surgery, Guangxi Medical University Cancer Hospital, Nanning, Guangxi Zhuang Autonomous Region 530021, China

## Abstract

Different opinions exist on the relationship between the C-reactive protein-to-albumin ratio (CAR) and the prognosis of colon cancer. This study is aimed at evaluating the relationship between CAR and prognosis of stage II–III colon cancer and establishing a clinical prognosis model. Patients were randomised to a training set (566 cases) and validation set (110 cases). The relationship between CAR and clinicopathological variables was calculated, and the Kaplan-Meier method was used to analyse the overall survival (OS) rate of colon cancer. In the training set, colon cancer independent risk factors were included in the prognosis model and then tested in the validation set. The accuracy and discrimination of the model were assessed using the C-index and calibration curves. Compared with patients with low CAR, patients with high CAR showed significantly poorer survival (*P* = 0.020). In the multivariate analysis, CAR, carcinoembryonic antigen (CEA), lymph node metastasis, operation mode, and perineural invasion were identified as independent prognostic indicators and adopted to establish the prediction model. The C-index of the nomogram for predicting OS reached 0.751 in the training set and 0.719 in the validation set. The calibration curve exhibited good consistency. In the present study, the CAR may be an independent prognostic factor for stage II–III colon cancer, and the nomogram has a certain predictive value. However, further prospective large-sample research needs to be conducted to validate our findings.

## 1. Introduction

Colon cancer has the fourth highest incidence of all tumours and is the fifth leading cause of cancer deaths worldwide [1]. Clinical and pathological data, including age, sex, tumour site, AJCC TNM stage, and the number of lymph node dissection (LND), have been shown to correlate with survival outcomes. Thus, considerable attention has been paid to preoperative biomarkers and tumour prognosis evaluation. Systemic inflammatory biomarkers, such as PLR, NLR, and C-reactive protein-to-albumin ratio (CAR), have been recently reported to be closely related to a patient's prognosis in colorectal carcinoma (CC), but the relationship is not well defined [2–5]. Which systemic inflammatory biomarker is most associated with the prognosis remains unclear. Patients with stage I CC have a good prognosis; however, those with stage IV CC who also present with an inflammatory response carry tumours with more aggressive biological behaviour and poorer survival [6]. The relationship between the prognosis of stage II or III colon cancer and inflammatory factors is not well defined. In addition, a single factor does not seem sufficient to predict prognosis. Hence, our group determined that combining factors would allow better prognostic prediction. Because these biomarkers are easily obtained from routine preoperative examinations, they have attracted increasing attention. To our knowledge, this is the first time that our hospital has established a model to evaluate the prognosis of patients with CC.

A nomogram is a two-dimensional graphic calculator that can conveniently diagnose diseases and rapidly evaluate prognosis. In addition to the conventional AJCC standard, clinicians can even collect the medical history of colon cancer patients and pre-/postoperative laboratory tests to predict patients' death risk. The present study assessed the prognostic value of CAR in patients and established a life nomogram to predict the 1- and 3-year overall survival (OS) rate of patients with stage II–III colon cancer.

## 2. Materials and Methods

### 2.1. Database and Candidate Variables

All patients underwent tumour resection and were diagnosed with colon cancer after the operation. Patients who met the following inclusion criteria were enrolled: (1) postoperative pathological diagnosis confirmed adenocarcinoma, (2) performance of resection for colon tumours, (3) patients undergoing limited operation, (4) follow-up at least 6 months after the operation, and (5) patients with stage II–III colon cancer. The exclusion criteria were as follows: (1) patients undergoing emergency operation, (2) patients with preoperative radiotherapy or chemotherapy, (3) history of tumour surgery, (4) patients with palliative operation, and (5) clinical diagnosis of hereditary nonpolyposis colorectal cancer or familial adenomatous polyposis clinical diagnosis of hereditary nonpolyposis colorectal cancer or familial adenomatous polyposis. Obstruction is defined as the inability of the colonoscope to pass through the intestine and the dilatation of the proximal intestine. All procedures were conducted following the ethical standards declared by the medical association. Given the retrospective nature of the study, the requirement of informed consent was waived, and patient data were kept confidential. This study was approved by the Institutional Review Board and Independent Ethics Committee of Cancer Hospital to Guangxi Medical University. The variables selected in this paper include clinicopathological data and biomarkers (Table 1). All 566 patients have complete follow-up records, including telephone, e-mail, SMS, and WeChat.

### 2.2. Determination of Variable Cutoff

The survival receiver operating characteristic (ROC) curve was used for statistical analysis by IBM SPSS 26.0 software. The point that lies closest to the upper left-hand corner of the graph is chosen as the cutoff. The cutoff point of CAR was 0.14.

### 2.3. Univariate and Multivariate Analyses

The following variables for univariate associations with OS were analysed: (1) clinical and pathological data: age (continuous), sex (male/female), BMI (continuous), tumour site (left/right), postoperative chemotherapy (absent/present), LNDs (continuous), operation mode (open/laparoscopic), intestinal obstruction (absent/present), ASA grade (1/2/3), preoperative comorbidity (absent/present), T-stage (T1–2/T3/T4), lymph node metastasis (LNM; absent/present), lymphovascular invasion (absent/present), and perineural invasion (PNI) (absent/present) and (2) laboratory markers: CEA (continuous), CA199 (continuous), CAR (≥0.14/<0.14), PLR (continuous), and NLR (continuous). Finally, variables that showed statistical significance at *P* value < 0.05, including CAR, CEA, LNDs, LNM, and PNI, were used in multivariable modelling.

### 2.4. Model Construction and Validation

We built a training set with 566 patients. Multivariate Cox proportional hazards models of OS were formulated from all variables and two-way interactions, showing statistically significant correlations with their respective endpoints. If a variable's influence has a clinical difference in the level of interaction, it will reach clinical significance. The final model, including all significant and pairwise interactions, was still statistically significant (*P* < 0.05) and clinically significant after a backward stepwise method. On the basis of the final model, nomograms (calculators) of 1- and 3-year OS probability were constructed with the R software package. We extracted 110 patients from the test data set to form a validation set. The performance of the nomogram model was evaluated in the validation set by examining calibration and discrimination. On the basis of multivariate Cox proportional regression analysis, the variables with *P* < 0.05 were used to establish a nomogram. In the internal calibration plots, points parallel to the reference line represent covariates with similar prediction results in the training and validation sets.

### 2.5. Statistical Analysis

The Kaplan-Meier method was used for survival analysis by IBM SPSS 26.0 software (version 26.0; SPSS, Chicago, IL). Statistical significance was set at 0.05. We developed the prognostic model with univariate predictive variables evaluating the significance of each clinicopathological biomarker. Next, multivariate analyses were performed using the Cox proportional hazards model. Univariate predictive variables with *P* < 0.05 were applied to multivariate analyses in order to identify the independent prognostic factors. Nomogram and calibration plots were constructed using R software, version 3.3.3 (CRAN; R Foundation for Statistical Computing, Vienna, Austria) [7].

## 3. Results

### 3.1. Patient Characteristics

After strict screening, 566 clinical cases were included in the training set, and 110 in the validation set. The median OS time was 36.2 months (range 6–130). The 6-month, 1-year, and 3-year follow-up rates were 100%, 87%, and 42%, respectively. The clinical data and laboratory results of all patients are summarised in Table 1.  Table 2 shows the relationship between CAR and clinicopathological parameters. CEA, tumour diameter, and preoperative PLR affected the preoperative CAR level. In the training set, the 6-month, 1-year, and 3-year OS rates after radical resection were 100%, 98%, and 87%, respectively (no survival chart is provided).

### 3.2. Univariate and Multivariate Analyses of Clinical Variables Associated with OS in the Training Set

**Figure 1:**
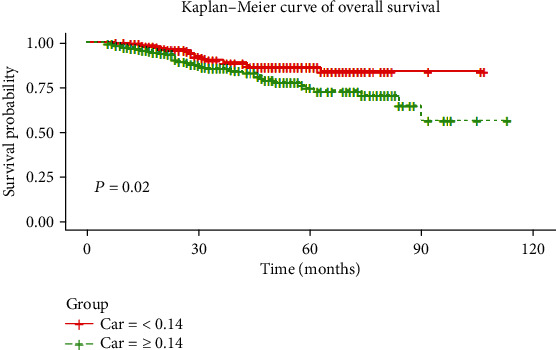
Kaplan-Meier curves for OS by the presence of CAR.

The relationships between variables and OS for colon cancer are listed in Table 3. In univariate analysis, OS was obviously related to the following factors: CAR (*P* = 0.022), CEA (*P* = 0.011), CA199 (*P* < 0.001), postoperative chemotherapy (*P* < 0.001), LNM (*P* < 0.001), operation mode (*P* = 0.002), LVI (*P* = 0.002), and PNI (*P* < 0.001). Patients with a lower CAR had a favorable prognosis for OS (Figure 1).

The results of the multivariate analysis of clinical and laboratory parameters correlated with OS are shown in Table 3. CAR (*P* = 0.002), CEA (*P* = 0.016), LNM (*P* < 0.001), operation mode (*P* = 0.041), and PNI (*P* = 0.012) were significantly associated with OS.

### 3.3. Construction and Verification of a Nomogram for OS

**Figure 2:**
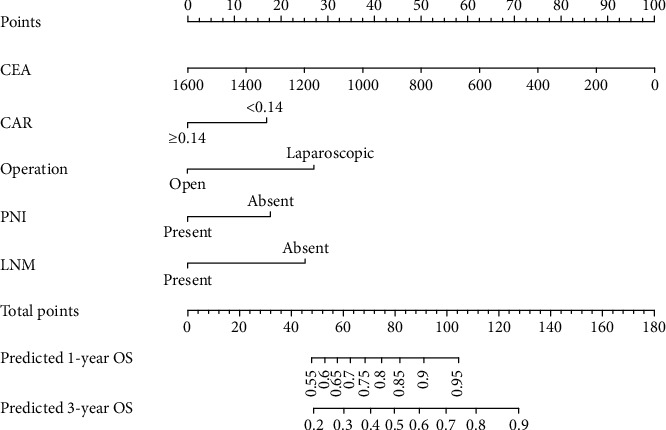
The life nomogram to predict OS rate after surgical resection for stage II-III colon cancer.

**Figure 3:**
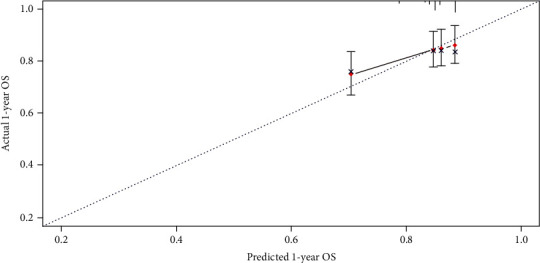
Calibration curves for 1-year prediction in the validation set.

**Figure 4:**
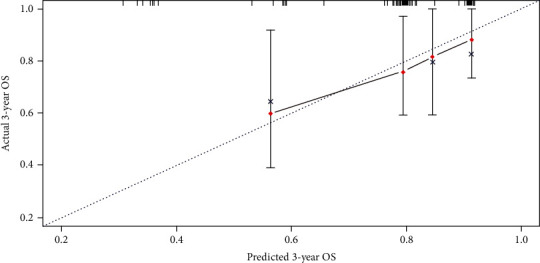
Calibration curves for 3-year prediction in the validation set.

Figure 2 shows a nomogram constructed by independent risk factors after multivariate Cox analysis for OS in the training set. In the training and validation sets, the nomogram's C-indices for prediction of OS were 0.751 and 0.719, respectively. For both sets, the calibration curves for 1- or 3-year OS exhibited high consistency between predicted values by the nomogram and the actual observations in the validation set (Figures 3 and 4).

## 4. Discussion

This is a single-centre study that evaluated the correlations between OS and 20 variables in stage II–III colon cancer patients after operation, including inflammation biomarkers and clinicopathological parameters. This study confirmed that CAR, CA199, CEA, operation mode, postoperative chemotherapy, LNM, LVI, and PNI were related to the prognosis in univariate analysis. Finally, CAR, CEA, LNM, operation mode, and PNI were independent prognostic indicators in multivariate analysis. Using the above variables, we established a visual nomogram model. Moreover, the nomogram has been well verified in the internal validation set. The calibration plot points are almost parallel to the reference line.

Inflammation biomarkers, including PLR, NLR, and CAR, play different evaluation roles and can predict the prognosis and complications in patients with radical colon cancer [8, 9]. The increase of preoperative CRP reflects the ongoing inflammatory reaction and is related to the decrease of lymphocytes, which leads to the impairment of cell-mediated immune function in patients with colorectal cancer [10]. In cancer patients, malnutrition or nutrition consumption usually shows hypoproteinaemia, which is related to an increased risk of postoperative infection complications [10, 11]. In studying the predictive factors, CAR showed to be valuable in the survival analysis of colon cancer. Given that CAR was a continuous variable, ROC curves were used to establish the CAR cutoff value in our study. We defended that this was the best way to create two groups (high and low CAR) for a given sample with suitable sensitivity and specificity levels. The cutoff point of CAR was much higher than those of Hashimoto et al. and Ishizuka et al. [12, 13]. One possible explanation was the inconsistent inclusion criteria, such as the TNM stage, age, and tumour site. In the present study, CAR is related to sex, tumour site, obstruction, PLR, NLR, and operation mode. The nomogram shows that other factors, such as age, CEA, and operation mode, weaken the weight of CAR in influencing prognosis. We also concluded that low CAR has a better OS than high CAR, similar to previously reported findings [9, 12, 14]. CAR is a routine clinical examination, which is easy to be obtained. It is expected to become a promising biomarker for evaluating the prognosis of colon cancer patients.

An elevated serum CEA level is commonly seen in patients with solid tumours, especially in the digestive tract. Similar to other studies [15–18], preoperative CEA levels were independent prognostic factors in stage II–III colon cancer in this present study, whilst elevated preoperative CEA levels were negatively correlated with prognosis. Moreover, increased postoperative CEA was related to the recurrence and distant metastasis of colorectal cancer [15, 19]. The latest AJCC guidelines did not regard preoperative CEA as a high-risk factor affecting stage II colon cancer prognosis. Spindler et al. suggested that elevated preoperative CEA is a high-risk feature of stage II colorectal cancer [15, 19]. Quah et al. identified that patients with elevated preoperative CEA should receive preoperative chemotherapy [20]. Unlike some research results, the CAR's cutoff value was 0.14 in the present study, which was obviously lower than those of some reported results [2, 15, 16]. Considering that CEA is a continuous variable, information loss will occur in variable data conversion. Hence, we did not assign the CEA value as a binary variable, which can reduce the prediction model's error.

Compared with open surgery, laparoscopic or robot-assisted colectomies were 1.5–2.5 times more likely to achieve adequate LNDs [21]. Studies have shown that the number of LNDs is related to survival rates. The long-term curative effect of the laparoscopic group was obviously better than that of the traditional open group. The reason may be explained by the decreased postoperative complication rates and blood transfusions with better preservation of the early postoperative cellular immune response in LCCS. Compared with open surgery, patients displayed higher CD8^+^ counts postoperatively and lower plasma levels of CRP [22]. Moreover, in an animal model study, the postoperative serum interleukin-6 in the laparotomy group increased significantly [23].

One hundred and fifty years ago, Cruveilheir first proposed the concept of tumour nerve infiltration. More and more studies emphasise the influence of PNI on prognosis. PNI is another way of colon cancer metastasis besides the blood supply and lymphatic metastasis and seems a reasonable risk factor. As a predictive and independent prognosis factor of the oncological outcomes in colon cancer, especially in stage II–III colon cancer, PNI was considered a high-risk factor for disease recurrence [24, 25]. Therefore, postoperative chemotherapy was strongly recommended for these patients to improve survival [26, 27]. In a previous study, the incidence of PNI was 56%, which was significantly higher than that reported by other authors [25, 28]. Including T4 (57%) colon may be the reason for this high value, and this also explains why we calculated the 3-year OS rate.

At present, standard clinical scoring systems usually use individual factors to evaluate prognosis risk. They have the advantage of simplicity and convenience. However, because each risk factor's weight is equal and the interaction of individual risk factors is not considered, their accuracy is not optimal. The influence of interaction between variables on prognosis is not considered. The nomogram helps to avoid these shortcomings. As a visual tool, it can provide more accurate prediction results for specific patients. In the occurrence, outcome, prognosis, and recurrence of diseases, a nomogram has rich clinical application value and is gradually adopted by clinicians.

In the nomograph, each variable corresponds to a point in the line. Then, the point is used as the intersection point perpendicular to the top line, which is the variable's fraction. Add up all the points, and the total score is the final predicted value. Clinicians can obtain each variable, so the prediction tool is practical and convenient. In this study, we investigated 20 variables of clinical and haematological parameters potentially related to prognosis. Amongst them, five variables could independently predict prognosis. Thus, the nomogram constitutes a cheap tool for evaluating the prognosis. In the internal verification data set, the nomogram's prediction accuracy and discrimination ability were well verified, indicating its excellent performance.

The shortcomings of this study still cannot be ignored. Firstly, selective bias exists in a single-centre retrospective study. Secondly, all laboratory parameters were only preoperative test results. Clinicians need to pay attention to the changes in preoperative and postoperative test results. Thirdly, because the nomogram model's establishment requires complete data, cases with incomplete data will be excluded, which may lead to selection bias. Finally, we did not validate the prediction model using external data, and we need to check our prediction model against external data in the future.

## 5. Conclusion

As a novel biomarker, CAR is an independent factor affecting stage II–III colon cancer and is negatively correlated with prognosis. The life nomogram model can help doctors make further treatment plans for colon cancer patients after surgery.

## Figures and Tables

**Table 1:** Clinicopathological characteristics of stage II-III colon cancer patients.

Variables		Training	Validation
Sex	Male	353	89
	Female	213	41
Age	<60	303	61
	≥60	263	49
Tumour site	Left colon	285	50
	Right colon	281	60
BMI (kg/m^2^)		21.3	21.0
Intestinal obstruction	Absent	463	82
	Present	103	28
ASA grade	1	295	54
	2	235	45
	3	36	11
Comorbidities	Absent	51	12
	Present	514	98
PLR		206	214
NLR		3.2	4.5
CAR	<0.14	242	41
	≥0.14	324	69
CEA (median, mg/ml)		15.6	15.4
CA199 (median, mg/ml)		38	29
Postoperative chemotherapy	Absent	124	14
	Present	442	96
Operation mode	Open	170	27
	Laparoscopic	396	83
LNDs		19	17
T-stage	T1-2	30	5
	T3	212	42
	T4	324	63
LNM	Absent	348	67
	Present	218	43
LVI	Absent	285	69
	Present	281	41
PNI	Absent	247	39
	Present	319	71

LND: lymph node dissection; LNM: lymph node metastasis; LVI: lymphovascular invasion; PNI: perineural invasion.

**Table 2:** Demographic and clinicopathological characteristics of stage II-III colon cancer patients with CAR.

Variables		Low CAR (<0.14)	High CAR (≥0.14)	*P* value
Sex	Male	136	217	0.009
	Female	106	107	
Age (median, year)	60 (16-89)	57 (16-88)	62 (19-89)	0.884
Tumour site	Left	131	154	0.120
	Right	111	170	
BMI (kg/m^2^)	21.3 (8.6-43)	21.2 (8.9-41.3)	21.3 (8.6-43)	0.366
Tumour diameter	5.4 (1-15)	5.4 (2-8)	5.4 (1-15)	<0.001
ASA grade	1	172	123	0.212
	2	122	113	
	3	15	21	
Comorbidities	Diabetes	23	21	0.225
	Pneumonia	10	18	
	Hypertension	22	26	
	Heart disease	3	4	
Intestinal obstruction	Absent	50	74	0.535
	Present	192	250	
PLR	206 (55-1113)	211 (55-355)	200 (134-1113)	<0.001
NLR	3.2 (0.53-196)	3.5 (0.57-193)	3.0 (0.53-196)	0.547
CEA (median, mg/ml)	3.5 (0.2-1500)	3.3 (0.2-1500)	5.4 (0.37-1000)	0.043
CA199 (median, mg/ml)	12 (0-1200)	11.2 (0.10-1200)	13.4 (0.00-1200)	0.672
Postoperative chemotherapy	Absent	209	254	0.015
	Present	33	70	
Operation mode	Open	55	115	0.001
	Laparoscopic	187	209	
LNDs	19 (1-80)	18 (1-80)	19 (1-56)	0.121
LNM	Absent	131	217	
	Present	110	107	
T-stage	T1-2	11	19	0.020
	T3	81	131	
	T4	150	174	
LVI	Absent	149	208	0.522
	Present	93	116	
PNI	Absent	98	149	0.192
	Present	144	175	

LND: lymph node dissection; LNM: lymph node metastasis; LVI: lymphovascular invasion; PNI: perineural invasion.

**Table 3:** Univariate analysis and multivariate analysis in the training set.

Variables		Univariate analysis			Multivariate analysis		
		HR	95% CI	*P* value	HR	95% CI	*P* value
Sex	Male	ref	—	—			
	Female	1.120	0.693-1.812	0.644			
Age (median, y)	59	0.996	0.978-1.014	0.658			
Tumour site	Left	ref	—	—			
	Right	1.066	0.663-1.712	0.793			
BMI (kg/m^2^)	21.3	1.001	0.964-1.039	0.957			
Tumour diameter	5.3	0.980	0.970-1.105	0.745			
ASA grade	1/2	ref	—	—			
	3	0.711	0.495-1.110	0.425			
Comorbidities	Absent	ref	—	—			
	Present	1.011	0.799-1.103	0.375			
Intestinal obstruction	Absent	ref	—	—			
	Present	0.666	0.395-1.120	0.125			
PLR	206	1.001	0.999-1.003	0.295			
NLR	3.2	1.002	0.976-1.028	0.906			
CAR	<0.14	ref	—	—	ref	—	—
	≥0.14	1.785	1.086-2.933	0.022	2.271	1.345-3.834	0.002
CEA (median)	0.2	1.002	1.001-1.004	0.011	1.002	1.001-1.003	0.002
CA199 (median)	11.99	1.002	1.001-1.004	<0.001			
Postoperative chemotherapy	Absent	ref	—	—			
	Present	2.561	1.584-4.140	<0.001			
Operation mode	Open	ref	—	—	ref	—	—
	Laparoscopic	0.580	0.360-0.934	0.025	0.601	0.368-0.980	0.041
LNDs	19	0.983	0.608-1.591	0.944			
LNM	Absent	ref	—	—	ref	—	—
	Present	2.561	1.584-4.140	<0.001	2.545	1.530-4.235	<0.001
T-stage	T1-2	ref	—	—			
	T3	0.879	0.625-1.237	—			
	T4	0.913	0.589-1.415	0.048			
LVI	Absent	ref	—	—			
	Present	2.178	1.341-3.538	0.002			
PNI	Absent	ref	—	—	ref	—	—
	Present	2.426	1.384-4.252	0.002	0.601	0.368-0.980	0.012

LND: lymph node dissection; LNM: lymph node metastasis; LVI: lymphovascular invasion; PNI: perineural invasion.

## Data Availability

The data that support the findings of this study are available from the corresponding author upon reasonable request.
